# Analysis of the sulfur-regulated control of the cystathionine γ-lyase gene of *Neurospora crassa*

**DOI:** 10.1186/1756-0500-5-339

**Published:** 2012-07-02

**Authors:** Brad S Reveal, John V Paietta

**Affiliations:** 1 Department of Biochemistry and Molecular Biology, Wright State University, Dayton, OH 45435, USA

**Keywords:** Cystathionine γ-lyase, Transsulfuration, Sulfur gene regulation, CYS3 regulator, *cys-16*^*+*^, *Neurospora crassa*

## Abstract

**Background:**

Cystathionine γ-lyase plays a key role in the transsulfuration pathway through its primary reaction of catalyzing the formation of cysteine from cystathionine. The *Neurospora crassa* cystathionine γ-lyase gene (*cys-16*^*+*^) is of particular interest in dissecting the regulation and dynamics of transsulfuration. The aim of this study was to determine the regulatory connection of *cys-16*^*+*^ to the *Neurospora* sulfur regulatory network. In addition, the *cys-16*^*+*^ promoter was characterized with the goal of developing a strongly expressed and regulatable gene expression tool.

**Findings:**

The cystathionine γ-lyase *cys-16*^*+*^ gene was cloned and characterized. The gene, which contains no introns, encodes a protein of 417 amino acids with conserved pyridoxal 5’-phosphate binding site and substrate-cofactor binding pocket. Northern blot analysis using wild type cells showed that *cys-16*^*+*^ transcript levels increased under sulfur limiting (derepressing) conditions and were present only at a low level under sulfur sufficient (repressing) conditions. In contrast, *cys-16*^*+*^ transcript levels in a Δ*cys-3* regulatory mutant were present at a low level under either derepressing or repressing conditions. Gel mobility shift analysis demonstrated the presence of four CYS3 transcriptional activator binding sites on the *cys-16*^*+*^ promoter, which were close matches to the CYS3 consensus binding sequence.

**Conclusions:**

In this work, we confirm the control of cystathionine γ-lyase gene expression by the CYS3 transcriptional activator through the loss of *cys-16*^*+*^ expression in a Δ*cys-3* mutant and through the *in vitro* binding of CYS3 to the *cys-16*^*+*^ promoter at four sites. The highly regulated *cys-16*^*+*^ promoter should be a useful tool for gene expression studies in Neurospora

## Findings

### Background

Cystathionine γ-lyase (E.C. 4.4.1.1; also known as γ-cystathionase) catalyzes the conversion of cystathionine to cysteine and α-ketobutryrate. Cystathionine γ-lyase, therefore, plays a key role in the transsulfuration reactions involved in the interconversion of homocysteine and cysteine routing through the intermediate cystathionine. Secondary reactions catalyzed include elimination reactions of homoserine, cystine, and cysteine; which in the latter case yield hydrogen sulfide (H_2_S). The proposed role of H_2_S as a physiologic signaling molecule has led to a number studies with the mammalian cystathionine γ-lyase [1,2], including gene knock-out constructs in mice [3]. Interestingly, the reverse transsulfuration pathway using cystathionine β-synthase and cystathionine γ-lyase, appears restricted to fungal and mammalian systems among eukaryotes [4,5]. Thus, the fungi offer an additional model system for cystathionine γ-lyase studies.

The properties and expression of cystathionine γ-lyase in fungi have been examined in several species. In *Neurospora crassa* the enzyme has been purified [6] and enzyme assays have shown up to a 30x increase in activity upon sulfur starvation [7,8] as well as low activity under sulfur sufficient conditions [8]. In *Aspergillus nidulans*, the cystathionine γ-lyase gene (designated *mecB*) has been cloned; *mecB* transcript levels show derepression under low sulfate levels; but showed increased transcription (with proportional enzyme activity increases) following growth with methionine supplementation [9]. In contrast, the *Acremonium chrysogenum* cystathionine γ-lyase gene does not appear to be regulated by the level of methionine [10]. In *N. crassa*, unlike these other fungi, the sulfur level provided for growth appears to have a straightforward effect on either derepressing or repressing cystathionine γ-lyase enzyme activity level [8].

The *N.* crassa sulfur regulatory system is composed of a genetically defined set of trans-acting regulatory genes and a set of structural genes encoding enzymes used in the uptake and assimilation of a variety of sulfur compounds [11-13]. Basically, when *N. crassa* is cultured under conditions of sulfur limitation (i.e., derepressing conditions) then an entire set of sulfur-related enzymes is coordinately expressed. The available data [8], therefore, suggests that *N. crassa* cystathionine γ-lyase is likely under control of this control system; along with other confirmed sulfur-regulated genes encoding arylsulfatase, sulfate permeases I and II, choline sulfatase, and others. The key regulator in the control system is the CYS3 bZIP transcriptional activator which is essential for sulfur structural gene expression [11]. The consensus binding sequence for CYS3 has been determined by *in vitro* binding-site selection studies [14].

Besides the cystathionine γ-lyase enzymatic assays done under sulfur-limiting and sulfur –sufficient conditions mentioned above, there is additional data available from microarray studies that have examined gene expression during *Neurospora crassa* development that include *cys-16*^*+*^ transcript levels. A major increase in *cys-16*^*+*^ transcript level was observed during the initial phase of the conidial germination process [15]. Starting with dormant conidia, there was an approximately 2.5-fold increase in *cys-16*^*+*^ transcript level at 1 hour into the germination process [15]. The *cys-16*^*+*^ transcript level then slowly declined, from the peak level seen at 1 hour, over an incubation time of 16 hours to end up slightly below the starting level (i.e., that seen in dormant conidia). The elevation in *cys-16*^*+*^ transcript (and presumably cystathionine γ-lyase activity) seems likely to represent an accession of the available cystathionine pool in order to supply the cellular demand for cysteine as germination commences and active growth begins. Consistent with this idea, additional microarray studies demonstrated a 3.6-fold higher *cys-16*^*+*^ transcript level in the active growth periphery (i.e., hyphal tips) as compared to a colonies interior region (i.e., 12–15 hours old) [16]. A final note regarding available microarray data is that a 2-fold increase in *cys-16*^*+*^ transcript level was observed post-exposure to phytosphingosine (which induces programmed cell death and notable increases in transcript level for many metabolism–related genes) [17]. The microarray data provides a useful developmental context for future studies of *cys-16*^*+*^.

In this report, we present the cloning, transcript and promoter analysis of the *N. crassa* cystathionine γ-lyase (*cys-16*^*+*^) gene. The data extend the role of the CYS3-directed regulatory system to include the transsulfuration pathway through the control of cystathionine γ-lyase and provide an additional model system to study the dynamics of transsulfuration. In addition, the tight regulation and high expression level of the *N. crassa* cystathionine γ-lyase gene make it’s promoter a potentially valuable tool for studies requiring manipulated control of gene expression.

### Results and discussion

#### Sequence and characterization of the cystathionine γ-lyase gene

**Figure 1:**
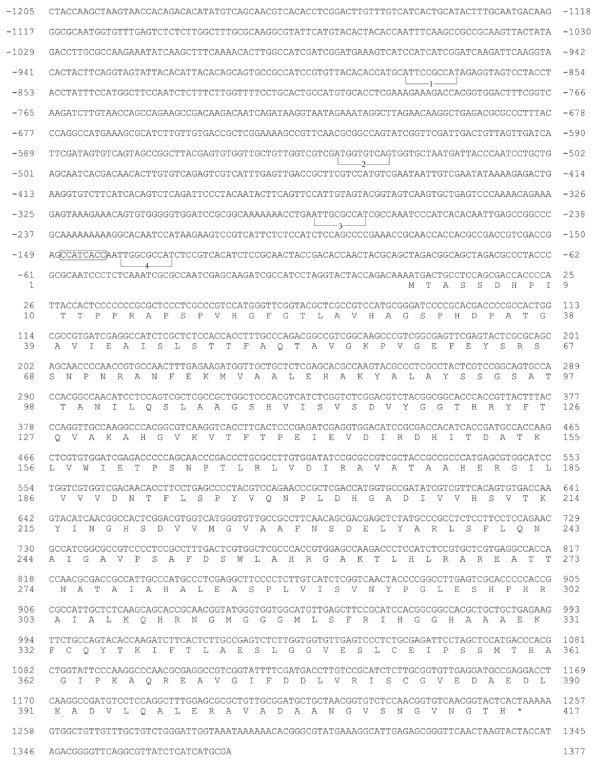
**Nucleotide and predicted amino acid sequence of the *cys-16***^***+ ***^**gene.** The sequence is shown from 1205 nucleotides upstream of the translation start codon to 125 nucleotides downstream of the stop codon (indicated by an asterisk). The nucleotides are numbered relative to the initiator ATG codon. CYS3-binding sites within the *cys-16*^*+*^ promoter are bracketed and numbered 1 through 4. The sequence corresponding to the *N. crassa* transcriptional initiation site consensus is boxed.

The nucleotide sequence of the *cys-16*^*+*^ gene and flanking 5’ region is presented in Figure 1. The cloning of *cys-16*^*+*^ used a cDNA clone, designated as N-EST:Nc3A10, with homology to cystathionine γ-lyases that had been isolated by the Neurospora Genome Project [18]. The Nc3A10 clone was used to probe a λ-J1 *N. crassa* genomic library by plaque hybridization and the identified clone λ used to generate subclones for nucleotide sequencing. The *cys-16*^*+*^ gene, as reported here, was originally isolated prior to the sequencing of the complete *N. crassa* genome and the sequence submitted as GenBank AF401238. Based on cDNA and genomic sequence data the *cys-16*^*+*^ gene has no introns. A putative transcriptional start CCATCACC is located at −140 upstream of the initiator ATG. Four CYS3 binding sites that were identified in this study (described below) are also annotated in Figure 1. The *cys-16*^*+*^ gene encodes a polypeptide of 417 amino acids with a highly conserved pyridoxal 5’-phosphate binding site and substrate-cofactor binding pocket [19]. *N. crassa* cystathionine γ-lyase shows substantial similarity to fungal (e.g., 77% identity to *Aspergillus nidulans* XP_659050 and 60% identity to *Saccharomyces cerevisiae* AAC04945)and to human (46% identity,NP_001893) cystathionine γ-lyases.

#### Analysis of *cys-16*^*+*^ gene expression

**Figure 2:**
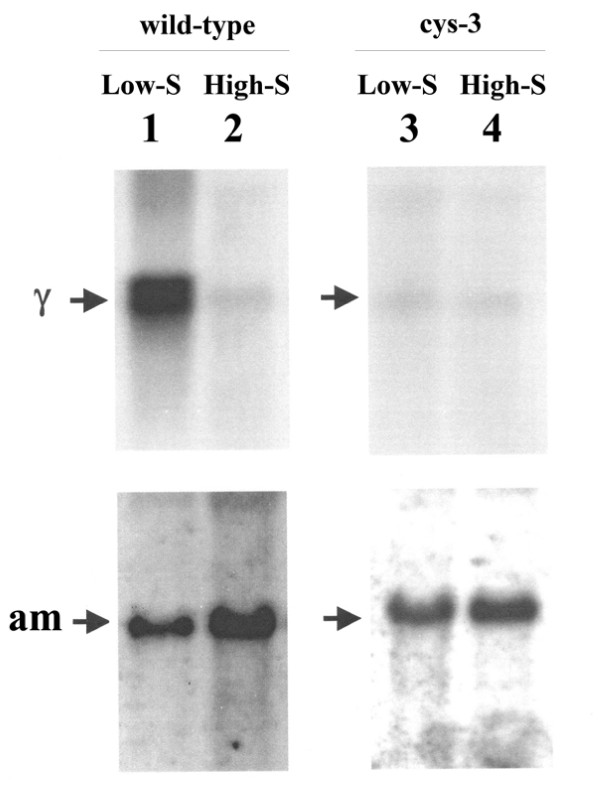
**Northern blot analysis of *cys-16***^***+ ***^**expression.** (Left panel) Northern analysis of poly (A)^+^ mRNA from wild-type *N. crassa* grown under low sulfur (or derepressing) (lane 1) and high sulfur (or repressing conditions) (lane 2). Northern blots were prepared probed with ^32^P-labeled *cys-16*^*+*^ DNA (designated γ within figure) and *am*^*+*^ DNA (which served as a control to ensure comparability between samples). Note high level of *cys-16*^*+*^expression under low sulfur conditions. (Right panel) Northern analysis of poly (A)^+^ mRNA isolated from the Δ*cys-3* (18–4) strain of *N. crassa* grown under low sulfur (or derepressing) (lane 3) or high sulfur (or repressing) (lane 4) conditions. Blots were probed as above. Note low level expression of *cys-16*^*+*^ for both low and high sulfur levels.

*cys-16*^*+*^ mRNA size and level were first assayed in *N. crassa* wild-type grown on low and high levels of sulfur (i.e., derepressing and repressing conditions, respectively) . Poly(A)^+^ mRNA was isolated, and Northern blots prepared and probed with the cloned *cys-16*^*+*^ gene. For the blot shown in Figure 2, mRNA was isolated from cultures grown for 12 h in low- and high sulfur media (i.e., low- or high-levels of supplemented methionine) inoculated with wild-type conidia. A 1.6-kb message hybridized to the *cys-16*^*+*^ probe and showed a high level of expression under low sulfur (derepressing) growth conditions. In contrast, the *cys-16*^*+*^ transcript was detectable at only a low level under high sulfur (repressing) growth conditions. The constitutively expressed glutamate dehydrogenase (*am*^*+*^) gene was used as a control for ensuring comparability of message levels between the high- and low-sulfur samples. These results suggest that in *N. crassa*, part of the complex response to sulfur limitation (or starvation) involves the derepression of *cys-16*^*+*^ gene expression and consequential increase in cystathionine γ-lyase activity converting the available pool of cystathionine into cysteine. The dynamics of how transsulfuration is regulated in *N. crassa* appears to be different from other fungal species such as *A. nidulans *[9] and *A. chrysogenum *[10], where cystathionine γ-lyase levels increase under high methionine or are not regulated by methionine level, respectively.

The role of CYS3 in the regulation of *cys-16*^*+*^ was examined using Northern blot analysis of *cys-16*^*+*^ expression in a strain deleted for the *cys-3*^*+*^ gene (Δ*cys-3*). Northern blots of poly(A)^+^ mRNA isolated from Δ*cys-3* grown under low- and high-sulfur conditions were probed with the *cys-16*^*+*^ gene (Figure 2). The level of *cys-16*^*+*^transcript detected was equivalently low for both low- and high-sulfur growth conditions; and similar to the repressed level seen in wild-type under high sulfur growth conditions. The Δ*cys-3* mutant completely blocks the derepression of *cys-16*^*+*^ expression seen under low-sulfur growth conditions in wild-type. In this regard, the *cys-16*^*+*^ gene is showing the typical response of genes previously confirmed to be part *N. crassa* sulfur control circuit (e.g., arylsulfatase, sulfate permease I and II, choline sulfatase, and others) in that sulfur starvation derepresses transcription in wild-type and mutating the CYS3 regulator abolishes that derepression [11,13].

#### Gel mobility shift analysis of CYS3 regulator binding

**Figure 3:**
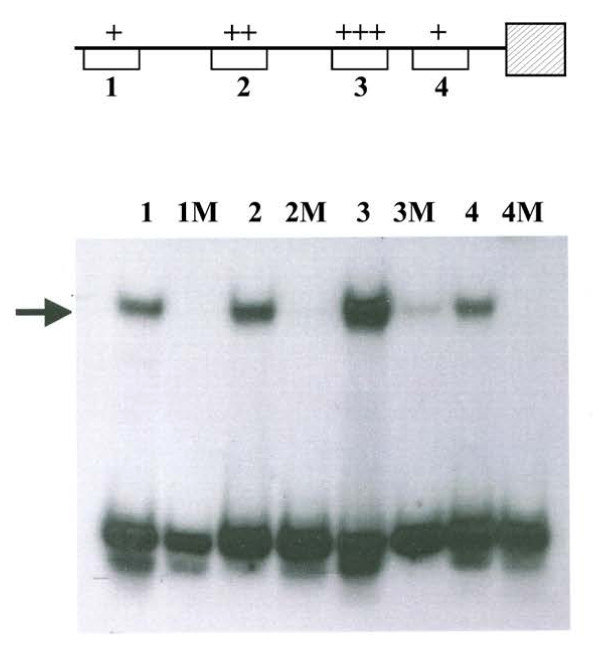
**Gel mobility-shift analysis of CYS3 binding. Lanes**: 1, CYS3-binding site 1 (nt −829 to −838); 1 M, mutated CYS3-binding site 1; 2, CYS3-binding site 2 (nt −531 to −540); 2 M, mutated CYS3-binding site 2; 3, CYS3-binding site 3 (nt −271 to −280); 3 M, mutated CYS3-binding site 3; 4, CYS3-binding site 4 (nt −128 to −137); 4 M, mutated CYS3-binding site 4. A single nucleotide within each CYS3 binding site was mutated to determine the specificity of CYS3 binding. The arrow indicates DNA fragments exhibiting reduced electrophoretic mobility due to protein-DNA interactions. The affinity of CYS3 for each binding site is depicted in the schematic of the *cys-16*^*+*^ promoter (top) (+, weakest affinity; +++ strongest affinity).

In order to further confirm the role of CYS3 in the control of *cys-16*^*+*^ expression, gel mobility shift assays were used to scan the *cys-16*^*+*^promoter for the presence of CYS3 binding sites. First, the *cys-16*^*+*^ promoter was divided into 100- to 300-bp segments by restriction endonuclease digestion and analyzed for CYS3 binding with a total of four short segments (24 bp) capable of binding CYS3 being defined (Figure 3). Each short segment contains a core 10 bp sequence that agrees strongly with the consensus binding site for CYS3 (5′ ATG GCGC CAT 3′) determined by *in vitro* binding-site selection [14]. Within the four detected binding sites, a gradation of CYS3 binding affinity in the gel shift assays is present, with CYS3 binding most strongly to site 3 (Figure 3). Note that the core sequence of site 3, 5′ TTG GCGC CAT 3′, is the closest match to the consensus CYS3 binding sequence (5′ ATG GCGC CAT 3′). Correspondingly, site 1 was the weakest binding site with the poorest match to the consensus CYS3-binding site. To examine the specificity of CYS3 binding to the *cys-16*^*+*^ promoter sites, a single nucleotide at the sixth position within the 10 base core for each CYS3-binding site was mutated from conserved G to a T. The single base mutation eliminated or substantially reduced CYS3 binding at each site. These data also provide a further confirmation of the CYS3 binding-site as determined by *in vitro* binding-site selection [14]. The definition of CYS3 binding sites to the *cys-16*^*+*^ promoter also provides necessary information for using the *cys-16*^*+*^ promoter as gene expression tool in *N. crassa*.

### Conclusions

The cystationine γ-lyase gene, *cys-16*^*+*^, is tightly regulated and is derepressed upon growth of *N. crassa* under conditions of sulfur limitation. Taken collectively, the *in vivo* expression experiments using a Δ*cys-3* mutant and the *in vitro* CYS3 binding data strongly supports the CYS3 regulation of *cys-16*^*+*^ expression. These experiments provide a starting point for examining the dynamics of transsulfuration control in a model eukaryotic system. In addition, the *cys-16*^*+*^gene contains a potentially useful regulatable promoter for gene expression studies.

### Methods

#### Strains, plasmids and culture conditions

74OR23-1a was used as the wild-type (WT) for these studies. Δ*cys-3* (18–4) was constructed and described in a prior study [20]. The λ-J1 *N. crassa* genomic library and N-EST:Nc3A10 clone were obtained from the Fungal Genetics Stock Center (Kansas City, KS). Vogel minimal medium [21], with supplements as required, was used. *N. crassa* cultures were grown at 25^o^ C. Sulfur repression and derepression experiments were done by growth of mycelia on Vogel-minus-sulfur medium plus high sulfur (5.0 mM methionine) and low-sulfur medium (0.25 mM methionine) medium, respectively.

#### Gene cloning and sequencing

The *N. crassa* cDNA clone N-EST:Nc3A10 was used to produce a ^32^P-labeled probe and carry out plaque hybridization as described [22] to a *N. crassa* λ-J1 genomic library and identify hybridizing clones. A segment containing the *cys-16*^*+*^gene derived from an isolate designated λ412 was subsequently subcloned into pSPORT and subjected to automated sequencing (Cleveland Genomics; Cleveland, OH) by primer walking. The genomic sequence was submitted to GenBank as AF401238. Following the cloning of the gene, the gene symbol *cys-16*^*+*^ was assigned by A. Radford (University of Leeds) and included in The *Neurospora crassa* e-Compendium [23]. The gene has also since been given the locus designation NCU09230 in the Broad Institute *Neurospora crassa* database [24].

#### Northern analysis

Poly(A)^+^ mRNA was isolated by phenol extraction and subsequent oligo (dT)-cellulose chromatography as described previously [20]. Briefly, mycelial samples were harvested by filtration, frozen in liquid nitrogen, and homogenized in a 1:1 mixture of phenol-chloroform-isoamyl alcohol (49:49:2) and extraction buffer (1% sarkosyl, 100 mM sodium acetate, 1 mM EDTA [pH 5.0]). After phenol-chloroform extractions, precipitation, and sodium acetate washes, and the poly(A)^+^ mRNA was isolated by oligo (dT)-cellulose chromatography. ^32^P-labeled probes were prepared by oligolabeling of DNA fragments [25]. Northern blots were hybridized and washed as outlined elsewhere [20].

#### Gel mobility shifts

Purified promoter fragments derived from restriction endonuclease digestion were used in an initial scan for CYS3 binding. Consequently, oligonucleotides representing the binding sites to be tested were synthesized (Applied Biosystems 391EP synthesizer), labeled by T4 polynucleotide kinase with [γ-^32^P]ATP, annealed and gel purified as described [14,26]. The following oligonucleotides (and complementary strands) representing the four putative CYS3 binding sites on the *cys-16*^*+*^ promoter were prepared: Site 1 [5′ ACCATGCATTCCGCCATAGAGGTA 3′], Site 2 [5′ GTCGTCGATGGTGTCAGTGGTGCT 3′], Site 3 [5′ AACCTGAATTGCGCCATAGCCAAA 3′], and Site 4 [5′ TCACCAATTGGCGCCATCTCCGTC 3′]. The synthesized mutated versions had a G to T substitution at the sixth position of the 10 bp core of the CYS3 consensus binding site: Site 1 M [5′ ACCATGCATTCC**T**CCATAGAGGTA 3′], Site 2 M [5′ GTCGTCGATGGG**T**TCAGTGGTGCT 3′], Site 3 M [5′ AACCTGAATTGC**T**CCATCGCCAAA 3′], and Site 4 M [5′ TCACCAATTGGC**T**CCATCTCCGTC 3′]. DNA-binding assays were carried out as we have described previously using *Eschericia coli* produced CYS3 protein [14,20]. Specificity of binding was ensured by control experiments using competition by addition of excess unlabeled DNA. Four percent PAGE gels with a 50 mM Tris-80 mM glycine-2 mM EDTA (pH 8.5) running buffer were electrophoresed at 20 mA with the temperature maintained at 4^o^ C. Quantitation of gel shift assays was performed by using a Molecular Dynamics Phosphorimager.

#### Availability of supporting data

The sequence data supporting the results of this article is available in the GenBank repository [AF401238, http://www.ncbi.nlm.nih.gov/nuccore/AF401238].

## Competing interests

The authors declare that they have no competing interests.

## Authors’ contributions

BR carried out the majority of experiments. JP and BR designed the experiments and prepared the manuscript. Both authors read and approved the final manuscript.
